# A Novel Hyaluronidase from Brown Spider (*Loxosceles intermedia*) Venom (Dietrich's Hyaluronidase): From Cloning to Functional Characterization

**DOI:** 10.1371/journal.pntd.0002206

**Published:** 2013-05-02

**Authors:** Valéria Pereira Ferrer, Thiago Lopes de Mari, Luiza Helena Gremski, Dilza Trevisan Silva, Rafael Bertoni da Silveira, Waldemiro Gremski, Olga Meiri Chaim, Andrea Senff-Ribeiro, Helena Bonciani Nader, Silvio Sanches Veiga

**Affiliations:** 1 Department of Cell Biology, Federal University of Parana, Curitiba, Parana, Brazil; 2 Department of Clinical Pathology, Clinical Hospital, Federal University of Parana, Curitiba, Parana, Brazil; 3 Department of Structural, Molecular Biology and Genetics, State University of Ponta Grossa, Ponta Grossa, Brazil; 4 Catholic University of Parana, Health and Biological Sciences Institute, Curitiba, Parana, Brazil; 5 Department of Biochemistry, Federal University of São Paulo, São Paulo, Brazil; Federal University of Minas Gerais, Brazil

## Abstract

Loxoscelism is the designation given to clinical symptoms evoked by *Loxosceles* spider's bites. Clinical manifestations include skin necrosis with gravitational spreading and systemic disturbs. The venom contains several enzymatic toxins. Herein, we describe the cloning, expression, refolding and biological evaluation of a novel brown spider protein characterized as a hyaluronidase. Employing a venom gland cDNA library, we cloned a hyaluronidase (1200 bp cDNA) that encodes for a signal peptide and a mature protein. Amino acid alignment revealed a structural relationship with members of hyaluronidase family, such as scorpion and snake species. Recombinant hyaluronidase was expressed as N-terminal His-tag fusion protein (∼45 kDa) in inclusion bodies and activity was achieved using refolding. Immunoblot analysis showed that antibodies that recognize the recombinant protein cross-reacted with hyaluronidase from whole venom as well as an anti-venom serum reacted with recombinant protein. Recombinant hyaluronidase was able to degrade purified hyaluronic acid (HA) and chondroitin sulfate (CS), while dermatan sulfate (DS) and heparan sulfate (HS) were not affected. Zymograph experiments resulted in ∼45 kDa lytic zones in hyaluronic acid (HA) and chondroitin sulfate (CS) substrates. Through *in vivo* experiments of dermonecrosis using rabbit skin, the recombinant hyaluronidase was shown to increase the dermonecrotic effect produced by recombinant dermonecrotic toxin from *L. intermedia* venom (LiRecDT1). These data support the hypothesis that hyaluronidase is a “spreading factor”. Recombinant hyaluronidase provides a useful tool for biotechnological ends. We propose the name Dietrich's Hyaluronidase for this enzyme, in honor of Professor Carl Peter von Dietrich, who dedicated his life to studying proteoglycans and glycosaminoglycans.

## Author Summary

Accidents involving brown spiders (*Loxosceles* genus) are reported throughout the world. South and Southeast of Brazil are endemic areas for this spider. *Loxosceles* bites commonly trigger local signs as swelling, erythema, hemorrhage and the hallmark symptom: a dermonecrotic lesion with gravitational spreading. Systemic effects are less common; however, are implicated in more severe cases. Hyaluronidases are referred in several venoms as “spreading factors” due to their enzymatic activity upon extracellular components. This activity facilitates the permeation of other toxins through the victim's body. In fact, a previous study identified the activity of *L. intermedia* venom upon glycosaminoglycans which are abundant components in the extracellular matrix of many tissues. Disclosing a little more about the role of hyaluronidases within this venom, we investigated the activities of a recombinant hyaluronidase from *L. intermedia* venom. Dietrich's hyaluronidase, as it was designated, was produced as a recombinant protein. By performing a rabbit skin dermonecrosis assay using Dietrich's Hyaluronidase and a dermonecrotic toxin, we showed that Dietrich's Hyaluronidase increased the dermonecrotic area induced by the dermonecrotic toxin. Our results confirm that hyaluronidases are a “spreading factor” of *L. intermedia* venom.

## Introduction

Bites involving brown spiders are characterized by skin injuries at the venom inoculation site, including swelling, erythema, hemorrhage, dermonecrosis, and the hallmark of loxoscelism: gravitational spreading of cutaneous lesions [1], [2]. Systemic involvement has also been reported including fever, malaise, weakness, nausea, vomiting and in severe cases, intravascular coagulation, hemolysis and acute renal disturbance [1], [2], [3], [4], [5].

The gravitational spread of skin lesions is a distinct characteristic of loxoscelism, described after experimental venom exposure in the skin of rabbits and in real cases. It appears hours or days after venom inoculation. Macroscopically, the development of lesions disperses in a gravitational direction with erythema, swelling, dark blue-violet color, and an eschar. Histologically, the lesion is reported as a collection of inflammatory cells in and around the blood vessels and diffusely distributed in the dermis. It is also possible to observe degeneration of blood vessel walls, disorganization of collagen fibers with edema, hemorrhage into the dermis, necrosis of cells, and destruction of tissue structures. Pathologically, the wound is described as aseptic coagulative necrosis [1], [2], [6], [7], [8].

The molecular mechanism by which brown spider venom induces gravitational spreading of skin lesions and systemic involvement is not fully understood. A fundamental requirement for venom to induce local spreading of lesions and systemic involvement is the presence of venom components that are able to degrade tissue barriers. The delivery of venom toxins to neighboring bite sites and into systemic circulation is assisted by molecules that degrade extracellular matrix constituents such as proteases and hyaluronidases [9], [10], [11], [12].

The venom is a mixture of proteins enriched in molecules with low molecular mass in the range of 5–40 kDa. Toxins including hyaluronidase, proteases, low molecular mass insecticidal peptides, Translationally Controlled Tumor Protein (TCTP) and phospholipases-D have been identified [1], [2], [13], [14], [15], [16], [17].

The existence of hyaluronidases in *Loxosceles* venoms comes from a previous report by Wright et al. (1973) [18], which reported hyaluronidase activity in the venom of *L. reclusa*. Additionally, hyaluronidase activity was described in the venom of *L. rufescens* [19] and several other *Loxosceles* venoms, including *L. deserta*, *L. gaucho*, *L. intermedia*, and *L. laeta* [11], suggesting biological conservation and significance of these enzymes. Da Silveira and colleagues (2007) [20] identified the hyaluronidases in *Loxosceles intermedia* venom as endo-β-N-acetyl-D-hexosaminidases that degrade hyaluronic acid and chondroitin sulfate. The idea that brown spider venom hyaluronidases play a role in the gravitational spreading of dermonecrosis and/or systemic diffusion of venom toxins, and then act as “spreading factors” comes from its degradative activity on hyaluronic acid and other glycosaminoglycans that mediate tissue integrity and stability. By degrading glycosaminoglycans, the hyaluronidase reduces the viscosity of hyaluronic acid and renders the extracellular matrix less rigid. This change makes the matrix more permeable to other toxins and facilitates the spread of other venom constituents and inflammatory cell mediators [21], [22].

Although hyaluronidase activity has been described in several venoms including that of snakes [10], [23], scorpions [24] spiders [22], bees [25], caterpillars [26], wasps [27], cone snails [28] and fish [29], the biochemical and biological characterization of these enzymes is restricted to a few examples [10], [21], [30]. In the case of the brown spider, the major technical problem is the minute volume of venom obtained per animal. The total volume harvested after an electric shock on the cephalothorax of the spider is limited to one or two microliters, and contains a few tens of micrograms of protein [31]. Purification of this native glycosidase to homogeneity for additional biochemical determination has not yet described for brown spider venom, and would be a very difficult task.

In recent years, using molecular biology techniques, scientists have obtained sufficient amounts of various recombinant toxins from brown spider venom to bring deeper insight into the molecular action of these toxins. Various venom phospholipase D isoforms and an Astacin-like metalloprotease have been reported for various species of spider venom [20], [32], [33], [34], [35], [36].

Here, by using a cDNA library of *L. intermedia* venom glands [15], we described the cloning, heterologous expression and functional evaluation of a novel hyaluronidase. The results bring insight into loxoscelism, opening up possibilities for biotechnological applications for this recombinant enzyme as a research tool. This recombinant hyaluronidase would also be useful in future structural and functional studies of this class of enzymes.

## Methods

### Reagents

Salts and organic acids were purchased from Merck (Darmstadt, Germany). Agar, β mercaptoethanol, molecular mass markers and purified hyaluronic acid were purchased from Sigma (St. Louis, USA). Ethidium bromide, a Wizard Plus SV miniprep kit and pGEM-T vector were acquired from Promega (Madison, USA). Agarose, IPTG and Trizol were purchased from Invitrogen (Carlsbad, USA). We acquired DNA molecular mass standards, X-Gal, Taq DNA polymerase, Pfu DNA polymerase, T4 DNA ligase, restriction enzymes, dNTPs and CIAP from Fermentas (Burlington, Canada). For bacterial culture, we used tryptone, yeast extract and agar purchased from HiMedia (Mumbai, India). The antibiotics were purchased from USB (Cleveland, USA). The bacterial strains used in this study and the ImPromII Reverse Transcription System kit were acquired from Invitrogen. We purchased the pET-14b expression plasmid from Novagen (Novagen, Madison, USA). The glycosaminoglycan standards used were heparan sulfate from bovine pancreas [37], chondroitin sulfate from bovine cartilage and dermatan sulfate from pig skin (Seikagaku, Kogyo Co., Tokyo, Japan).

### cDNA library construction and screening

The venom gland cDNA library construction was performed as described by Gremski et al. (2010) [15]. Briefly, processed sequences were compared to GenBank sequences using the *Basic Local Alignment Search Tools blastx*, *blastn* (E values<1e-05) and *tblastx* (E values<1e-10) algorithms. Afterwards, ESTs were manually inspected for functional classification. One toxin-coding messenger similar to a hyaluronidase from *Rattus novergicus* (gb|EDL77243.1) was found in this cDNA library and was used as a base sequence.

### Amplification of the cDNA 5′ end

To obtain the complete 5′ end of hyaluronidase cDNA, a 5′RACE (Rapid Amplification of 5′cDNA Ends) protocol was performed following Sambrook and Russel (2001) [38] with minor modifications. Briefly, 1 µg total RNA from *L. intermedia* venom glands was used as a template. The first-strand cDNA was synthesized using the gene-specific reverse primer R1 (5′-GTTGCAGGGTAGACAACATCCACG-3′) and the Improm-II Reverse Transcriptase (Promega), according to the manufacturer's instructions. The cDNA was recovered by ethanol precipitation in the presence of ammonium acetate. The cDNA was poly(A) tailed with terminal deoxynucleotidyl transferase (Fermentas), as recommended by the supplier. The modified cDNA was amplified using PCR with a (dT)_17_ adaptor primer (5′-CGGTACCATGGATCCTCGAGTTTTTTTTTTTTTTTTTV-3′) and the nested gene-specific reverse primer R2 (5′-CTCCATGCTTCCCAGTCGATGATGC-3′) using a Pfu DNA polymerase (Fermentas). Finally, the PCR product was purified from the gel using Illustra GFX PCR DNA and a Gel Band Purification kit (GE) following the manufacturer's instructions and sequenced on both strands using MegaBace DNA Analysis Systems (Amersham Bioscience).

### Amplification of the cDNA 3′end

To achieve the complete sequence, a 3′RACE (Rapid Amplification of 3′cDNA End) protocol was modified from Sambrook and Russel [38]. Briefly, 1 µg total RNA from *L. intermedia* venom glands was used to synthesize the first-strand cDNA using the gene-specific forward primer F1 (5′-CGAATCAATCAACGGTGGCATCCCTC-3′) and the Improm-II Reverse Transcriptase (Promega). The cDNA was recovered as previously described. The cDNA was amplified with F2 (5′ -CCGCATTGGTTTTAGCCGCATTC-3′), (dT)_17_ adaptor primer (5′ -CGGTACCATGGATCCTCGAGTTTTTTTTTTTTTTTTTV-3′) and Pfu DNA polymerase (Fermentas). Purification from the gel with a Gel Band Purification kit (GE) was performed according to manufacturer's instructions, and the amplicon was sequenced on both strands.

### Recombinant protein expression

The cDNAs encoding the putative mature hyaluronidase, which we named Dietrich's Hyaluronidase, were amplified with PCR using primers designed to contain *Nde* I restriction sites at the 5′ ends (5′-GGAATTCCATATGGACGTCTTCTGGAACG-3′) and *BamH* I sites (5′-CGGGATCCCTCACTTTGTTTTCTGCTC-3′). The PCR product was digested with *Nde* I and *BamH* I restriction enzymes. Subcloning was performed with a pET-14b plasmid (Novagen) digested with the same enzymes. The recombinant construct for mature protein was expressed as a fusion protein with a 6× His-Tag at the N-terminus. The expression construct was inserted into *E. coli* BL21(DE3)pLysS cells and plated on LB-agar plates containing 100 µg/mL ampicillin and 34 µg/mL chloramphenicol. Single colonies of the construct were inoculated into LB broth (100 µg/mL ampicillin and 34 µg/mL chloramphenicol) and grown overnight at 37°C. These cultures were diluted 1∶100 into 1 L fresh LB broth/ampicillin/chloramphenicol and incubated at 37°C until the OD_550 nm_ = 0.4–0.6. Recombinant protein expression was induced by the addition of 0.1 mM IPTG (isopropyl β-D-thiogalactoside) and cells were incubated for 3.5 h at 30°C in a shaker. Cells were harvested by centrifugation (4,000×g, 7 minutes, 4°C), suspended in 20 mL of extraction buffer (50 mM sodium phosphate pH 8.0, 500 mM NaCl, 10 mM imidazole, 1 mg/mL lysozyme) and frozen at −20°C overnight.

### *In vitro* refolding of Dietrich's Hyaluronidase

Cells were thawed and disrupted with 8 cycles of sonication at medium intensity for 20 seconds using a 500-W ultrasonic cell disruptor. Lysed materials were centrifuged (20,000×g, 30 minutes, 4°C), and the pellet was washed with a denaturing buffer (100 mM Tris-HCl pH 10.0, 2 M urea, 1% Triton X-100) and sonication at low intensity. After centrifugation at 6,000×g for 10 minutes the resulting pellet was solubilized in 100 mM Tris-HCl pH 10.0 with 8 M urea and 100 mM DTT. The solution containing denatured and reduced recombinant protein was adjusted to a concentration of ∼5 mg/mL and was added dropwise (1∶10 ratio) to a refolding buffer (100 mM Tris-HCl pH 10.0, 3 mM reduced glutathione, 0.3 mM oxidized glutathione, 0.4 M L-arginine, 0.2 mg bovine serum albumin) by stirring over 16 h at 4°C. The protocol used was based on the works of Burgess et al. and Hofinger and co-workers [39], [40]. Dialysis was against phosphate buffered saline, and recombinant hyaluronidase was concentrated using filter devices (MWCO 30,000 Millipore, Schwalbach, Germany).

### SDS-PAGE and immunoblotting

Polyclonal antibodies against *L. intermedia* whole venom and against recombinant hyaluronidase were produced in rabbits as described by Harlow and Lane (1988) [41], with minor modifications [42]. Protein concentration was determined using the Coomassie Blue method [43] or ultraviolet measurement (280 nm). For protein analysis, 12.5% SDS-PAGE was performed under reducing conditions and gels were stained with Coomassie Blue. For immunoblotting, proteins were transferred onto a nitrocellulose membrane following Towbin et al. (1979) [44] and immunodetected using hyperimmune antisera, which reacts with hyaluronidase or venom.

### Specificity of Dietrich's Hyaluronidase activity upon glycosaminoglycan substrates

Agarose gel electrophoresis was developed in 50 mM Tris–acetate buffer at pH 8.0 to evaluate hyaluronic acid degradation. Electrophoresis was performed in 50 mM 1,3-diaminopropane acetate buffer, pH 9.0 (Aldrich, Milwaukee, USA) to evaluate the cleavage activity of glycosidase on chondroitin sulfate, dermatan sulfate and heparan sulfate. After electrophoresis, compounds were precipitated in the gel using 0.1% cetavlon (cetylammonium bromide) for 2 h at room temperature. The gels were dried and stained with Toluidine Blue. The glycosaminoglycan standards used were as previously described [20], [45]

### Ethics statement

The Ethics Animal Experiment Commitee of the Setor de Ciências Biológicas of the Federal University of Parana, established by the decree 787/03-BL from May 9th 2003, and upon the internal regiment, certifies that the procedures using animal in this work are in agreement with the Ethical Principals established by Experimental Animal Brazilian Council (COBEA), and with the requirement of the “Guide for the Care and Use of Experimental Animals (Canadian Council on Animal Care)”. Processes numbers: 23075.052088/2008-32 Approved: April 7, 2009 and 23075.087106/2011-01 Approved: August 9, 2011.

### Animals

Adult rabbits (∼3 kg) from the Central Animal House of the Catholic University of Parana were used for *in vivo* experiments with whole venom and recombinant enzymes (the product of antibodies and dermonecrosis studies). All procedures involving animals were carried out in accordance with Brazilian Federal Law, following the Ethical Subcommittee on Research Animal Care from the Federal University of Parana.

### Dermonecrosis and gravitational spreading

To evaluate the potential gravitational spreading effect of brown spider hyaluronidase, 10 µg of a recombinant dermonecrotic toxin (LiRecDT1) diluted in PBS was injected intradermally into a shaved area of rabbit skin with or without recombinant hyaluronidase (10 µg). For the same purpose 10 µg of Dietrich's Hyaluronidase alone (diluted in PBS with 0.2 mg/mL BSA) was also injected. We used two negative controls: one was PBS with 0.2 mg/mL BSA, to assure that BSA used to refold recombinant hyaluronidase did not induce changes. The other was a recombinant protein with similar molecular mass obtained under the same conditions as hyaluronidase, but without hyaluronidase activity. The last assay guarantees that potential bacterial contaminants did not influence the results. Ten micrograms of venom and dermonecrotic toxin were used as positive controls. Rabbits were used in dermonecrosis experiments because this model reproduces skin lesions very close to those observed in accidents with humans [46]. Experiments were repeated with 4 animals and the development of experimentally induced dermonecrosis was observed 3 h, 6 h and 24 h after the injection.

### Histological methods for light microscopy

Rabbit skin pieces from animals that intradermally received recombinant proteins were collected following anesthetization with ketamine (Agribands, Campinas, Brazil) and acepromazine (Univet, São Paulo, Brazil). Collected tissue samples were fixed in “ALFAC” (ethanol 85%, formaldehyde 10% and glacial acetic acid 5%) for 16 h at room temperature. After fixation, samples were dehydrated in a graded series of ethanol before paraffin embedding (for 2 h at 58°C). Thin sections of 4 µm thickness were processed for histological procedures [42]. Tissue sections were stained with Masson's trichrome (TM) as described [47].

### Bioinformatics tools

To compare new sequences generated against the GenBank database, we used the BLAST site (http://blast.ncbi.nlm.nih.gov/Blast.cgi). The parameters for blastn were refined by selecting the “others” search set with E values <1e-05. For blastx, we chose standard genetic code 1 and non-redundant protein sequences with E values <1e-05 parameter. Deduced amino acid sequence was found using the Open Reading Frame Finder site (http://www.ncbi.nlm.nih.gov/projects/gorf/) and an analysis of protein parameters and glycosylation modifications was conducted using the ExPASy Bioinformatics Research Portal (http://web.expasy.org/protparam/). Disulfide bond prediction was made using the DiANNA 1.1 web server http://clavius.bc.edu/~clotelab/DiANNA/. CLUSTAL W (http://www.ebi.ac.uk/Tools/msa/clustalw2/) was used for alignment and cladogram production. Finally, to search any possible signature recognition within Dietrich's Hyaluronidase sequence, we used the InterProScan site (http://www.ebi.ac.uk/Tools/pfa/iprscan/).

### Genbank accession number

The Dietrich's Hyaluronidase cDNA sequence has been submitted to the Genbank database under accession number JX402631.

## Results

### Cloning of a hyaluronidase from the *L. intermedia* venom gland

**Figure 1:**
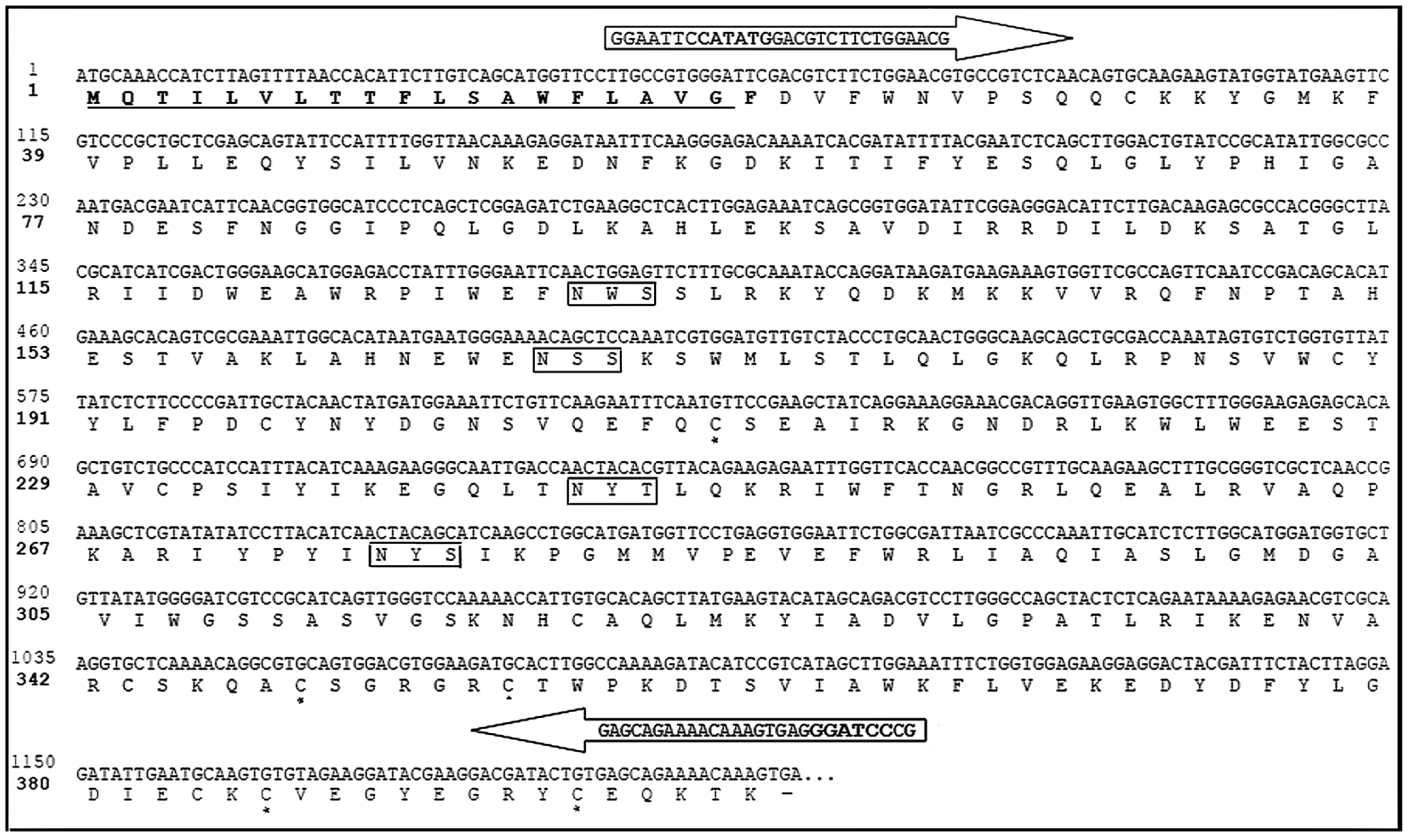
Molecular cloning of Dietrich's Hyaluronidase, a novel protein from *L.* intermedia venom gland. Nucleotide- and amino-acid-deduced sequences. Predicted amino acid sequence, with 400 amino acid residues and signal peptide underlined. Rectangular boxes are the predicted N-glycosylation sites (ExPASy tools parameters), and asterisks indicate cysteine residues that may constitute disulfide bonds (DiANNA web server). Arrows indicate primers with restriction sites (bold nucleotides) used for subcloning into pET 14b.

By screening clones of a cDNA library of the *L. intermedia* venom gland, a cDNA encoding for a hyaluronidase was obtained through a blastx search [15]. The putative protein product from this cDNA was designated Dietrich's Hyaluronidase (in honor and memoriam of Professor Carl Peter von Dietrich, born in 1936, deceased in 2005). The complete cDNA sequence of Dietrich's Hyaluronidase consist of 1200 bp with a single open reading frame (ORF) coding for 400 amino acids and a putative N terminal endoplasmic reticulum import signal of 19 residues. At least two types of post translational modification were observed: 4 putative N-glycosylation sites and 3 possible disulfide bonds (Fig. 1). The predicted molecular mass for mature Dietrich's Hyaluronidase protein was approximately 44.8 kDa, and its pI was 8.75.

### Multiple alignment analysis of the cDNA-deduced amino acid sequence and the cladogram relationship of Dietrich's Hyaluronidase to other hyaluronidase family members

**Figure 2:**
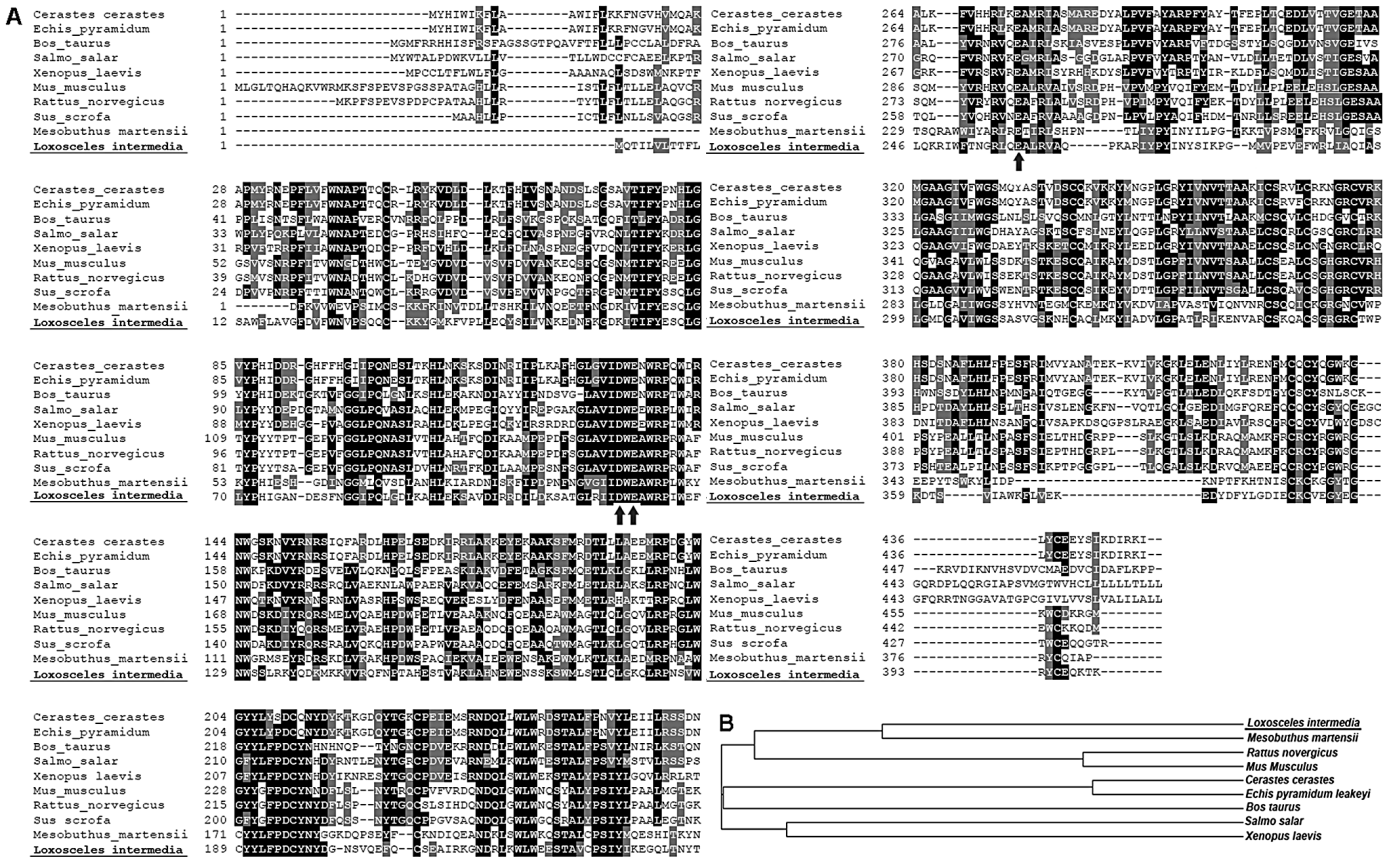
Multiple alignment analysis of Dietrich's Hyaluronidase sequence and several others hyaluronidases. (A) Deduced amino acid sequences compared and their GenBank accession numbers: *Cerastes cerastes* (ABI33939.1); *Echis pyramidum leakey* (ABI33941.1); *PH-20 Bos taurus* (AAP55713.1); Hyaluronidase-2 precursor *Salmo salar* (ACI33947.1); lysosomal hyaluronidase *Xenopus laevis* (AAL55823.1); hyaluronoglucosaminidase 1 *Mus musculus* (AAC15949.1); hyaluronidase 1 *Sus scrofa* (ACH56985.1); venom hyaluronidase *Mesobuthus martensii* (ACY69673.1); hyaluronoglucosaminidase 1 *Rattus norvegicus* (EDL77243.1) and *Loxosceles intermedia* (JX40263.1). Black shaded regions show amino acid identity and gray shaded regions, conservative substitutions. Arrows indicate conserved amino acids that appear to be important for catalytic hydrolysis. (B) Deduced cladogram of the cloned hyaluronidase and family members based on sequence alignment and percent identity generated by CLUSTAL W.

To explore the structural and evolutionary relationships among the newly identified glycosidase and other members of the hyaluronidase family, a BLAST GenBank database search through alignment of cDNA-deduced amino acid sequences revealed that Dietrich's Hyaluronidase has structural similarity to other hyaluronidase family members (Fig. 2A). The overall identity of hyaluronidase from *L. intermedia* venom is approximately 46% compared with the scorpion venom hyaluronidase of *Mesobuthus martensii* (gb|ACY69673.1). Dietrich's Hyaluronidase is also similar to snake venom hyaluronidases, sharing 33% sequence identity with *Echis pyramidium* (*gb|ABI33941.1*) and *Cerastes cerastes (gb|ABI33939.1)*. Interestingly, the enzyme from an arthropod venom showed approximately 30% amino acid identity with hyaluronidases from mammal species such as *Bos taurus (gb|AAP55713.1)* and *Rattus novergicus (gb|EDL77243.1)* (Fig. 2B). An InterProScan search matches Dietrich's Hyaluronidase with more than 500 proteins belonging to the glycoside hydrolase 56 family. A typical signature domain for hyaluronidases has not yet been demonstrated, and there are few studies about of the residues involved in catalysis [30], [48]. However, the hyaluronidase from *L. intermedia* venom has 3 conserved amino acids (indicated in Fig. 2A at arrows: D118, E120, E259) that appear to be important for the catalytic activity of some hyaluronidases [30].

### Expression, purification and refolding of Dietrich's Hyaluronidase

**Figure 3:**
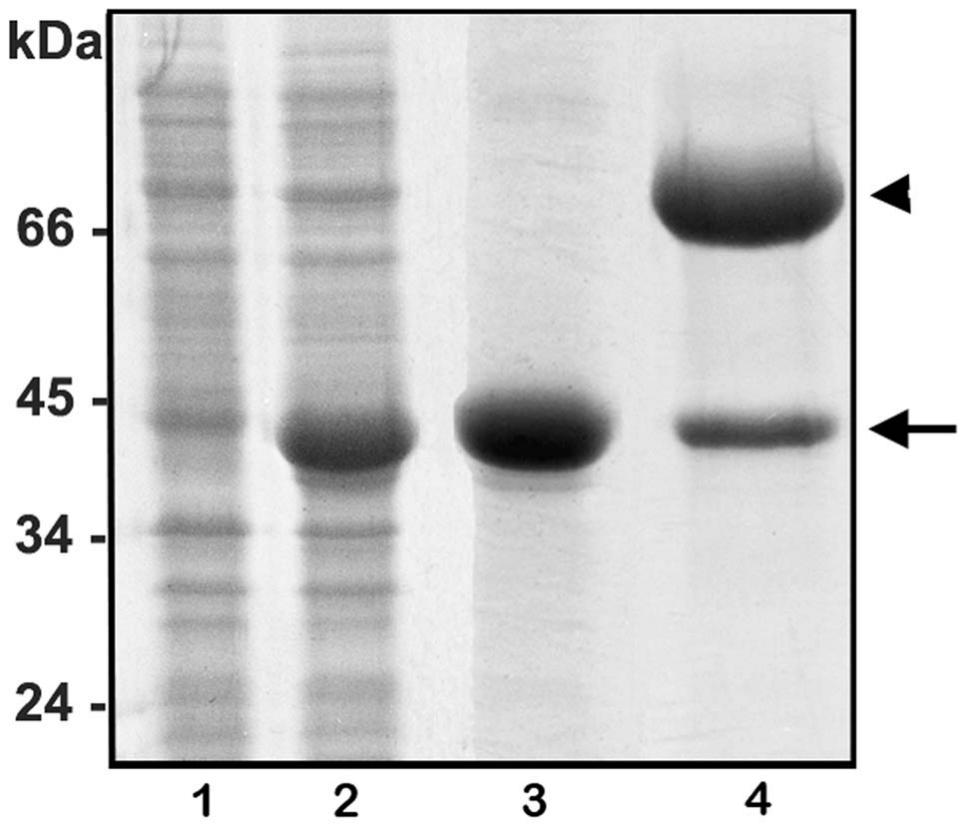
Heterologous expression, purification and refolding of Dietrich's Hyaluronidase. Expression of recombinant protein in *E. coli* BL21(DE3)pLysS was analyzed via 12.5% SDS-PAGE under reducing conditions and Coomassie blue staining. Lanes 1 and 2 depict *E. coli* before (0 h) and after induction (4 h) with 0.1 mM IPTG at 30°C, respectively. Lane 3 shows inclusion bodies after washing in denaturing conditions. Lane 4 shows recombinant hyaluronidase refolded *in vitro* at ∼45 kDa (arrow). Band at 67 kDa is BSA used during refolding (arrowhead). Molecular mass marker positions are shown in the left of figure.

Recombinant hyaluronidase from *L. intermedia* venom was expressed in pET 14b in *E. coli* BL21(DE3)pLysS cells. The expression of recombinant protein was optimized when induced for 3.5–5 h with 0.1 mM of IPTG. The recombinant protein was detected only in the insoluble fraction of cell lysates and was purified under denaturing conditions by washing inclusion bodies until there were no bacterial contaminants visible in SDS-PAGE gels stained with Coomassie blue. The SDS-PAGE mobility of recombinant protein reduced by β-mercaptoethanol treatment was ∼45 kDa, consistent with the calculated molecular mass (Fig. 3). After testing several refolding protocols *in vitro* (data not shown), the protocol described in the methods section, was effective in solubilizing recombinant hyaluronidase from inclusion bodies, as shown in lane 4 (Fig. 3).

### Immuno cross-reactivity among native and recombinant hyaluronidases

**Figure 4:**
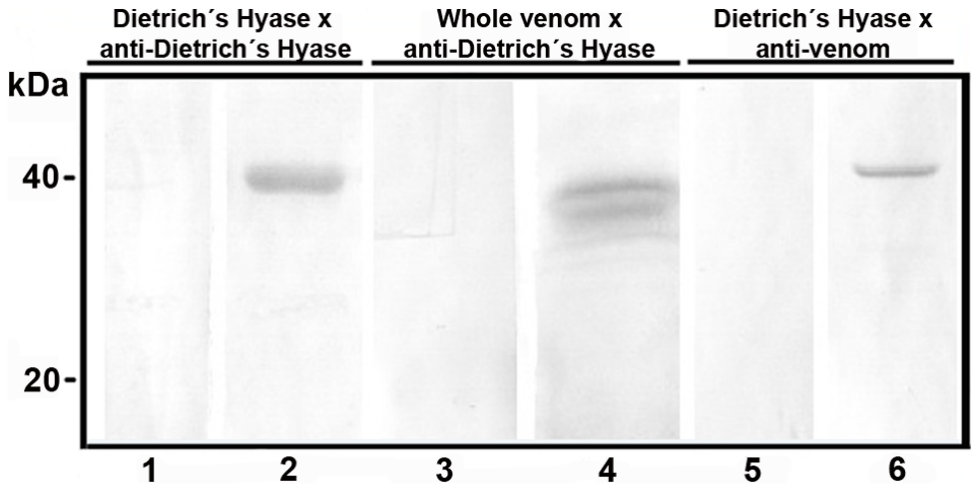
Immunological cross-reactivity of whole venom and Dietrich's Hyaluronidase. Purified recombinant glycosidase (2.0 µg; lanes 1, 2, 5 and 6) or whole venom (40 µg; lanes 3 and 4) were separated by 12.5% SDS-PAGE under reducing conditions, transferred onto nitrocellulose membranes and exposed to antibodies against Dietrich's Hyaluronidase (lanes 2 and 4) or whole venom toxins (lane 6). Lanes 1, 3 and 5 indicate reactions in the presence of pre-immune sera (control for antibody specificity). Molecular mass marker positions are shown in the left of figure.

Immunoblot analysis using antibodies against recombinant hyaluronidase (Dietrich's Hyaluronidase) and antibodies against whole venom toxins established the epitope relationship of native hyaluronidase with recombinant enzyme (Fig. 4). Antibodies against Dietrich's Hyaluronidase recognize hyaluronidase in the whole venom, as well as anti-whole venom serum reacted with recombinant glycosidase.

### Hyaluronidase activity on HA and CS

**Figure 5:**
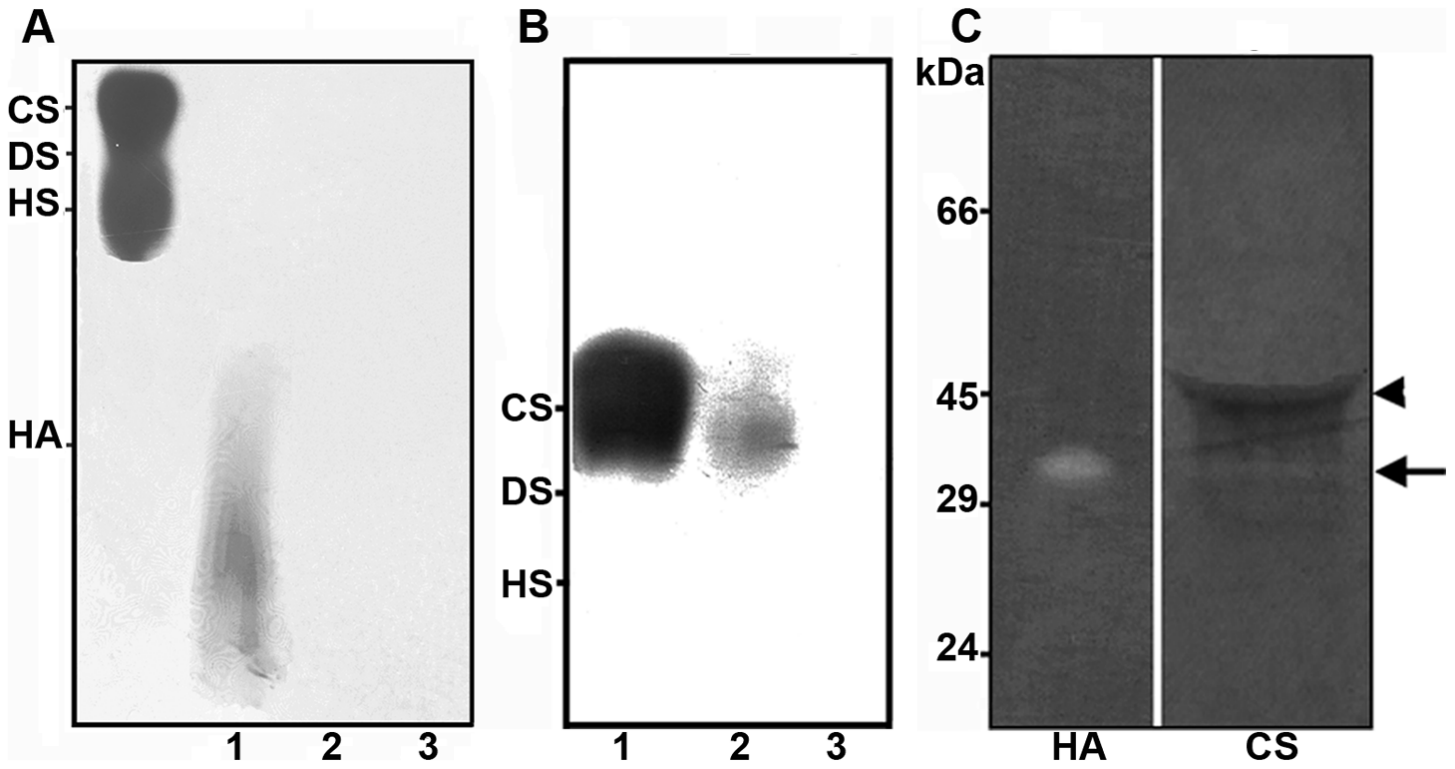
Detection of degradative activities of Dietrich's Hyaluronidase upon hyaluronic acid and chondroitin sulfate. (A) Purified hyaluronic acid (15 µg) was incubated with whole venom or recombinant hyaluronidase at a ratio of 1∶1 for 3 h at 37°C. Degradation was assayed after agarose gel electrophoresis of the incubation mixture, followed by cetavlon precipitation and toluidine blue staining of the glycosaminoglycan sample. Chondroitin sulfate (CS), dermatan sulfate (DS) and heparan sulfate (HS) were used as molecular mass markers. Lane 1 represents the negative control (15 µg of purified hyaluronic acid), and lanes 2 and 3 show hyaluronic acid incubated with the venom and recombinant hyaluronidase, respectively. (B) Purified chondroitin sulfate (CS) was incubated with venom (lane 2) or Dietrich's Hyaluronidase (lane 3) at ratios of 1∶1 for 16 h at 37°C. Chondroitin sulfate standard is shown in lane 1. (C) 5 µg of recombinant enzyme was submitted to zymography SDS-PAGE 10% impregnated with 0.17 mg/mL hyaluronic acid (HA) or 0.34 mg/mL chondroitin sulfate (CS). The zymogram was developed overnight at 37°C under pH 7.4 and the gel was stained with Alcian Blue and co-stained with Coomassie Blue. Black arrow indicates activity of recombinant hyaluronidase in zymograms. Arrowhead shows persistent BSA residue after proteolysis performed before coloration. Molecular mass marker positions are shown in the left of figure.

To evaluate the activity of Dietrich's Hyaluronidase after refolding, purified HA was incubated at a ratio of 1∶1 with recombinant hyaluronidase for 3 h, 6 h and 16 h at 37°C. The enzymatic activity was analyzed through agarose gel electrophoresis stained with toluidine blue. HA was completely degraded within 3 h (Fig. 5A, lane 3) showing that the *in vitro* refolding method was effective in adjusting hyaluronidase in an active conformation. Through analysis with CS, DS and HS at a substrate-to-enzyme ratio of 1∶1, we noted that recombinant hyaluronidase degraded CS (Fig. 5B, lane 3) and did not degrade DS and HS (data not shown), similar to the native hyaluronidases from venom.

To verify the stability of refolding *in vitro* achieved with recombinant hyaluronidase, we performed a zymogram assay using gels containing copolymerized purified HA and CS as substrates. Hydrolytic activity was found as a specific band between the 29–45 kDa regions in both substrates and strengthened the results described above (Fig. 5C).

### Gravitational spreading

**Figure 6:**
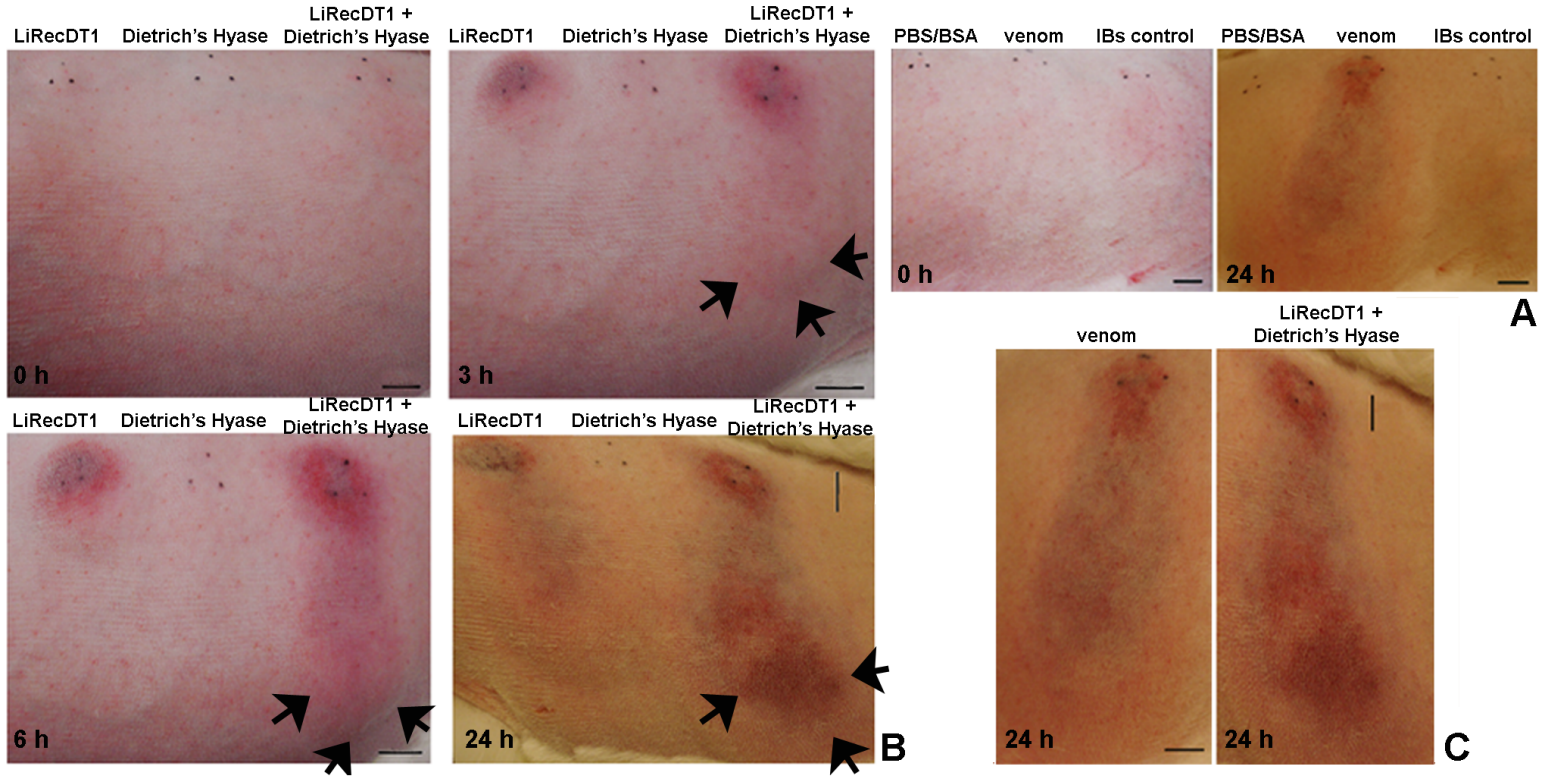
Macroscopic changes in rabbit skin exposed to whole venom, recombinant dermonecrotic toxin and Dietrich's Hyaluronidase. (A) Macroscopic visualization of skin injuries of rabbits intradermally injected with 10 µg of whole venom (as positive control), 10 µg of a recombinant protein not related to hyaluronic acid hydrolysis or dermonecrosis (IBs control) and PBS/BSA (negative controls). Lesions were observed 0 and 24 h following injection. (B) Macroscopic visualization of skin injuries of rabbits intradermally injected with 10 µg of dermonecrotic toxin (LiRecDT1), 10 µg of Dietrich's Hyaluronidase (Dietrich's Hyase), or both enzymes together (20 µg). Lesions were observed 0, 3, 6 and 24 h following injections. Arrows point to spreading of erythema (3 and 6 h), ecchymosis (6 and 24 h) and gravitational spreading of necrotic lesions (24 h) after injection of dermonecrotic toxin and Dietrich's Hyaluronidase, compared to lesions developed only due to dermonecrotic toxin. (C) In detail, comparison of dermonecrotic injuries developed due to venom or a mixture of recombinant hyaluronidase and phospholipase-D. Injections were applied in the center of the triangle indicated by three dots. Scale bar is shown at right of each picture and represents 1 cm.

We sought to develop dermonecrosis experiments using rabbit skin to determine the *in vivo* involvement of brown spider venom hyaluronidase in envenomation. Moreover, we aimed to observe the relationship of hyaluronidases in the skin deleterious activities of whole venom. Injections of *L. intermedia* crude venom, recombinant dermonecrotic toxin [33] (positive controls), and dermonecrotic toxin plus Dietrich's Hyaluronidase all induced macroscopic erythema, ecchymosis and dermonecrosis in rabbit skin, which was followed by 3, 6 and 24 h of observation (Fig. 6A and Fig. 6B). On the other hand, the injection of recombinant hyaluronidase alone, PBS/BSA and a recombinant protein not related to hyaluronic acid hydrolysis (negative controls) did not show macroscopic erythema, ecchymosis or dermonecrosis (Fig. 6A and Fig.6B). The length of dermonecrosis lesions in the rabbit skin of Dietrich's Hyaluronidase concomitantly injected with dermonecrotic toxin was substantially larger than for dermonecrotic toxin alone after 24 h (Fig. 6B). The macroscopic gravitational spreading and edema induced by the two recombinant enzymes injected together were very similar to whole venom (Fig. 6C).

### Histopathological findings of dermonecrosis

**Figure 7:**
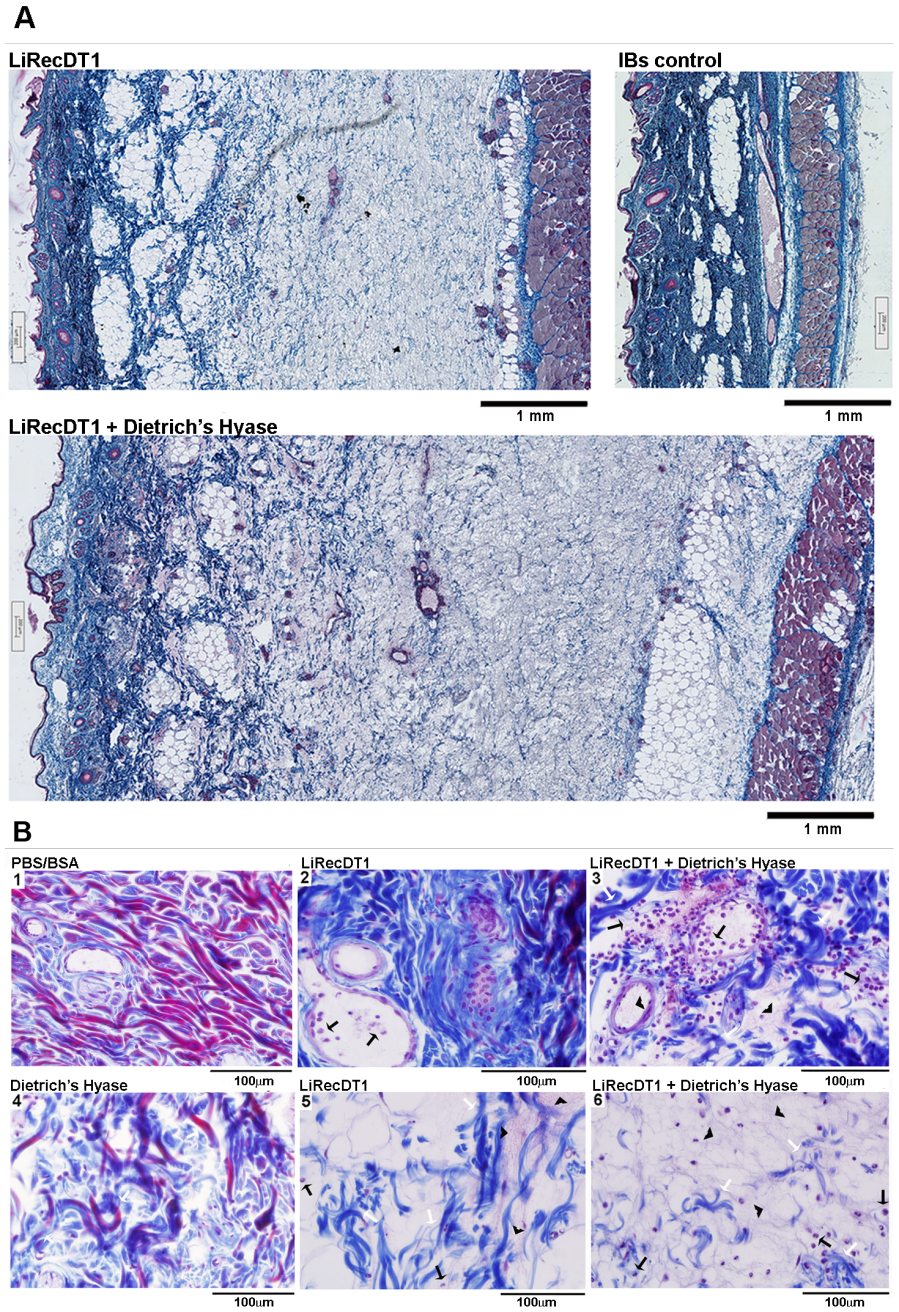
Histopathological changes in rabbit skin exposed to recombinant dermonecrotic toxin and Dietrich's Hyaluronidase. Light microscopic analysis of tissue sections was performed on rabbit skin after 24 h of recombinant enzymes injection. The tissue sections were stained with Massons's trichrome. The scale bar at the bottom of the figures indicates 1 mm (A) or 100 µm (B). (A) Edema triggered in rabbit skin by the combination of dermonecrotic toxin (LiRecDT1) and Dietrich's Hyaluronidase (Dietrich's Hyase), visualized by size. Comparison of the skin structures via scanning of images from epidermal (on the left of figure) to muscular tissues (on the right of figure) under the same laboratory conditions (magnification 15×). (B) Skin exposed to recombinant dermonecrotic toxin, recombinant hyaluronidase, or recombinant hyaluronidase plus recombinant dermonecrotic toxin changed the extracellular matrix in connective tissue: disorganization of collagen fibers (Fig. 7B-3, 7B-4, 7B-5 and 7B-6) (white arrows); fibrinoid exudate into the dermis (Fig. 7B-3, 7B-5 and 7B-6) (arrowheads); and intense inflammatory response with neutrophils inside blood vessels and diffusely into the dermis structures (Fig. 7B-2, 7B-3, 7B-5 and 7B-6) (black arrows) are shown (magnification 400×).

Light microscopic analysis of rabbit skin biopsies 24 h after of the experimentally induced dermonecrosis experiments performed above suggested that recombinant hyaluronidase was able to disorganize the extracellular matrix from rabbit skin dermis (Fig. 7B-4). Dermonecrotic toxin alone triggered typical inflammatory cell accumulation (Fig. 7B-2 and 7B-5) and edema signals (Fig. 7A, 7B-5), as well as collagen fiber disorganization (Fig. 7B-5) and presence of fibrin in connective tissue (Fig. 7B-5). Nevertheless, we noticed that these inflammatory events, developed by dermonecrotic toxin, were intensified when combined with Dietrich's Hyaluronidase (Fig. 7A; 7B-3 and 7B-6), supporting an extensive edema and spreading of lesion. By comparison, using a panoramic image under the same conditions, deleterious effects induced by dermonecrotic toxin alone were lower than those evoked by mixture of this toxin and Dietrich's Hyaluronidase (Fig. 7A). These microscopic findings corroborate the information obtained by macroscopic analysis of rabbit skin lesions.

## Discussion

Hyaluronidases are present in various tissues and are involved in important biological events, including embryonic development, inflammation, fertilization, tumor cell metastasis, bacterial pathogenesis and aging [10], [14]. These enzymes were described many years ago in the venom of different *Loxosceles* species, featuring a conservative event and suggesting biological significance in the life cycle of these spiders [1], [2], [11]. A more refined understanding of brown spider venom hyaluronidase is hampered by native molecules present in low amounts in the venom and by extremely minute volume of venom obtained from spiders [2], [15], [49].

Here, we described the cloning, heterologous expression, purification, refolding and characterization of a novel hyaluronidase from a cDNA library of the *L. intermedia* venom gland. These results corroborate previous biochemical data that have described these enzymes in brown spider venom from different species [11], [20]. The recombinant protein identified herein is designated Dietrich's Hyaluronidase (GenBank Accession no. JX402631).

The primary sequence of Dietrich's Hyaluronidase includes a hydrophobic signal sequence of 19 residues and a mature protein. The hydrophobic signal sequence probably directs its expression to the endoplasmic reticulum in venom gland epithelial cells. The molecular mass and isoelectric point calculated from the deduced amino acid sequence of mature protein were 44.8 kDa and pI 8.75, respectively. The theoretical molecular mass is within the expected range, considering the size of brown spider venom native hyaluronidases [11], [20], and the isoelectric point was similar to the calculated pI of hyaluronidases from other venoms. A protein sequence analysis of brown spider venom hyaluronidase compared to hyaluronidases from different sources showed that this venom enzyme has conserved amino acids (D118, E120, E259) that seem to be important for catalytic hydrolysis [30], [48]. Dietrich's Hyaluronidase was assigned to the Glycosidase 56 family; which compose a wide group of O-glycosyl-hydrolases [50]. The highest similarity to Dietrich's hyaluronidase was found in the sequences of scorpion hyaluronidase from *Mesobuthus martensii* venom (46% amino acid identity) and the snake venom of *Echis pyramidium* and *Cerastes cerastes* (33%). As might be expected, the hyaluronidase from *L. intermedia* venom was particularly similar to hyaluronidases from other venoms. However, we highlight Dietrich's Hyaluronidase's significant identity with mammalian species such as *Bos taurus* and *Rattus novergicus*. This fact, coupled with its activity at physiological pH, would be interesting for pharmaceutical use.

Dietrich's Hyaluronidase was heterogeneously expressed as a mature protein with an N-fusion His-tag using the *E. coli* BL21(DE3)pLysS strain. Isolation of the recombinant glycosidase was performed by washing inclusion bodies with denaturing buffer. The purification of recombinant proteins from inclusion bodies might be advantageous, because it is protease-resistant and is close to functional native structure [51], [52], [53].

Characterization of the antigenic cross-reactivity of recombinant hyaluronidase and *L. intermedia* venom showed that the venom contains proteins that have antigenic identity (sequence epitopes) with recombinant hyaluronidase because antisera raised against recombinant enzyme was able to react with native venom. Two protein bands of the whole venom were recognized by hyaluronidase antisera, corroborating with previous results that showed two lytic zones in zymography experiments using whole venom in a gel co-polimerized with hyaluronic acid. Those bands probably correspond to two isoforms of hyaluronidase present in *L. intermedia* whole venom [11], [20]. Additionally, anti-venom cross-reacted with the recombinant hyaluronidase, suggesting that the recombinant glycosidase retains linear antigenic determinants from native hyaluronidases. In this way, the antisera to Dietrich's Hyaluronidase or purified antibodies from it could be a possible biological tool in loxoscelism therapy or for research purposes.

The functionality of refolded recombinant hyaluronidase was demonstrated through its activity upon purified hyaluronic acid and chondroitin sulfate. Dietrich's Hyaluronidase was able to directly degrade hyaluronan and chondroitin sulfate. These results are in agreement with previous data reported for native hyaluronidases from whole venom, which degrade both glycosaminoglycans [20]. The hydrolytic activity found at 29–45 kDa in zymogram assays copolymerized with hyaluronic acid and chondroitin sulfate corroborates with the degrading activity of glycosaminoglycans viewed in agarose gels, suggesting certain stability in the active folding of Dietrich's Hyaluronidase acquired after refolding *in vitro*. Again, results show that recombinant hyaluronidase can also be considered a chondroitinase, as previously reported for native hyaluronidases [20].

Regarding *in vitro* refolding it is worth mentioning that buffers with different pHs, redox agents and additives were tested [39], [54] until the solubility and activity of Dietrich's Hyaluronidase was maximized (data not shown). When bovine albumin was removed from the buffer, the hyaluronidase activity was null. This result is consistent with the literature that describes how BSA may compete with hyaluronidases to form inactive electrostatic complexes with hyaluronic acid. This competition induces free hyaluronidase resulting in a large increase in the hydrolysis rate [55]. Several research groups have reported that serum proteins are able to enhance hyaluronidase activity and are sometimes required to detect the presence of hyaluronidases [55], [56], [57].

But what is the role of brown spider venom hyaluronidase on bite pathology? The involvement of hyaluronidase on the activity of brown spider venom is supported by the conservative phenomenon of this enzyme, which indeed is found in different species of *Loxosceles* venoms [11]. Hyaluronidases have been described for several venoms [26], [55], [58] and act as “spreading factors” by degrading hyaluronic acid and chondroitin sulfate. In this way, venom hyaluronidases may render surrounding regions at the bite site more permeable. Furthermore, these enzymes may facilitate the diffusion of other venoms constituents through the bodies of victims [10], [58]. A hyaluronidase isolated from funnel web spider venom was able to enhance the potency of a myotoxin and a hemorrhagic toxin. This work is in accordance with the hypothesis that hyaluronidase mediates enhanced toxicity of whole venom during envenomation [22].

A typical symptom of loxoscelism is the gravitational spreading of skin lesions that appear a few hours after bites or experimental envenomation of animal models [1], [2]. The mechanism by which *Loxosceles* venom causes gravitational spreading of dermonecrosis is not fully understood. Experimental inoculation of purified recombinant dermonecrotic toxins (phospholipases-D, non-proteolytic or hyaluronidase activities), evokes gravitational spreading of skin lesion on rabbits [34]. Macroscopically, Dietrich's Hyaluronidase increases the erythema, ecchymosis and dermonecrotic lesion area induced by recombinant phospholipase-D along exposure times observed. Histopathological findings for the dermonecrotic toxin exposed tissue samples revealed, as expected, the presence of a neutrophilic infiltrate, collagen fiber disorganization and signs of edema [36], [46]. However, when we analyzed tissue samples treated with phospholipase-D toxin plus Dietrich's Hyaluronidase, we observed that these inflammatory evidences were much more intense, as if recombinant hyaluronidase was allowed a free diffusion of phospholipase D by rendering the connective tissue more permeable. For the first time in the literature, experimental results strongly indicate that the hyaluronidase from *Loxosceles* venom is in fact a “spreading factor” for this venom.

For brown spider venom hyaluronidase, based on the spreading property, we extrapolated that this molecule would be primarily responsible for spreading other venom toxins from the bite site into the systemic circulation [1], [2]. Literature data have reported signs of systemic intoxication including fever, vomiting, hemolytic anemia, thrombocytopenia, disseminated intravascular coagulation, and nephrotoxicity following brown spider bites [1], [2], [4], [31].

Moreover, brown spider venom hyaluronidase is also suggested to play a role in the cutaneous lesions following bites, as described for other venom hyaluronidases [23]. By disturbing the extracellular matrix structure, venom hyaluronidase may influence the stability of blood vessel walls. These events may increase the spread of other venom toxins, which in turn can cause the cutaneous lesions that may follow after brown spider bites. Corroborating previous ideas, our work shows that Dietrich's Hyaluronidase is able to degrade hyaluronic acid and chondroitin sulfate *in vitro* and has increased the erythema and ecchymosis cause by phospholipase-D injected into rabbit skin. We suggest that this enzyme likely degrades both glycosaminoglycans *in vivo*. It is known that low molecular weight HA fragments are pro-inflammatory, immunostimulatory and angiogenic. Besides, HA oligosaccharides formed due to plasma HA degradation may result in hemostatic disturbances. Hence, exploring the *in vivo* fragmentation of HA and the effects on pathophysiology of envenomation might be a topic for further researches [59].

Another important feature that has been described for hyaluronidases of venoms, such as from scorpions, bees, hornets and wasps, is their classification as major allergens that can induce anaphylaxis and sometimes death [21], [60]. Based on its sequence identity with other venom hyaluronidases, it is possible that the brown spider venom hyaluronidase may act as a potential allergen in susceptible individuals. This notion is strengthened by evidence observed in the course of loxoscelism, which includes itch, morbilliform erythema, cutaneous rash and petechial eruption [1], [2], [31], [61], [62]. It might also suggest the involvement of the immune system [63], [64]. Cutaneous rashes respond to treatment with systemic steroids [31], [63]. Thus, the hyaluronidase from *Loxosceles* genus could be further investigated as a molecule capable of inducing allergy reactions. In the same way, whether allergy's molecular mechanism is due to the enzyme molecule itself or even the HA fragments resulting from its glycosidase activity.

Besides a novel understanding of the pathogenesis of loxoscelism, Dietrich's Hyaluronidase could be an important tool for future biotechnological purposes [14], [16]. Hyaluronidases are known to be involved in physiological and pathological processes such as bacterial pathogenesis, spread of toxins and venoms, fertilization, and cancer progression [65], [66], [67], [68].

For example, hyaluronidase recombinant molecules may be developed as tools for *in vitro* fertilization [10]. On the other hand, hyaluronidase inhibitors may serve as contraceptives, because they are involved in the fertilization of eggs by mammalian sperm and could thus be used to block fertilization. Other possible applications of hyaluronidase inhibitors are as anti-tumor [58], [66], anti-bacterial [67] and anti-venom/toxin agents [16], [20]. Interestingly, a cloned *Buthus martensi* hyaluronidase (BmHYA1) down-regulated CD44 (a hyaluronic acid-ligand transmembrane glycoprotein involved in cell–matrix connections) in a cancer cell line, suggesting that a variant of cancer cells can be modulated by external venom hyaluronidase treatment [24].

In summary, for the first time in the literature, we have described a recombinant hyaluronidase from the venom glands of *Loxosceles* sp. We cloned, expressed, purified and refolded this enzyme, which showed degradative activity on hyaluronic acid and chondroitin sulfate. This recombinant glycosidase increased the area of dermonecrosis, gravitational spreading and edema induced by a recombinant dermonecrotic toxin, mimicking the profile of whole venom in an animal model for skin loxoscelism *in vivo*. Finally, results showed the role of this enzyme as a spreading factor in the mechanism of spreading of necrotic lesions followed by brown spider envenomation. Together, these results provide insights into loxoscelism and contribute to a further understanding of venom mechanisms and will perhaps unveil novel treatment protocols for envenomation or biotechnological applications for this venom protein.

We propose naming this novel brown spider hyaluronidase described herein as Dietrich's Hyaluronidase, in memoriam and honor of Professor Carl Peter Von Dietrich. Professor Dietrich was born in 1936 in Rio de Janeiro, Brazil, graduated in medicine from the Universidade do Estado do Rio de Janeiro, 1963, Rio de Janeiro, Brazil, specialized in biochemistry at the Instituto de Investigaciones Bioquimicas, 1963, Buenos Aires, Argentine, and earned his doctorate at the University of Saskatchewan, 1970, Saskatchewan, Canada. He was a professor and researcher in biochemistry and molecular biology at the Universidade Federal de São Paulo, São Paulo, Brazil, where he worked with macromolecules such as proteoglycans, glycosaminoglycans and heparins. Professor Dietrich died in 2005 in São Paulo, Brazil.
